# Lichenoid and Granulomatous Stomatitis: Three Additional Cases and Detailed Immunohistochemical Analysis

**DOI:** 10.1111/cup.70183

**Published:** 2026-07-25

**Authors:** Mariana Paravani Palaçon, Karina Kendelhy Santos, Karina Helen Martins, Cecília Aparecida de Oliveira Campos, Ana Terezinha Marques Mesquita, Evânio Vilela Silva, Thales Peres Candido Moreira, Gabriel Enrique Tantalean Laor, Victor Hugo Toral‐Rizo, Alan Grupioni Lourenço, Heitor Albergoni Silveira, Jorge Esquiche León, Andreia Bufalino

**Affiliations:** ^1^ Department of Diagnosis and Surgery São Paulo State University (Unesp), School of Dentistry Araraquara SP Brazil; ^2^ Department of Dentistry, Clinical Stomatology Federal University of the Jequitinhonha and Mucuri Valleys Diamantina MG Brazil; ^3^ Oral Pathology, Department of Stomatology, Public Oral Health, and Forensic Dentistry, Ribeirão Preto Dental School (FORP/USP) University of São Paulo Ribeirão Preto SP Brazil; ^4^ Department of Pathology and Forensic Medicine, Ribeirão Preto Medical School (FMRP/USP) University of São Paulo Ribeirão Preto SP Brazil; ^5^ Department of Oral Pathology, School of Dentistry Autonomous University of the State of Mexico Toluca Mexico; ^6^ Department of Oral Diagnosis and Surgery State University of Northern Paraná (UENP) Jacarezinho PR Brazil

**Keywords:** immunohistochemistry, lichen planus, lichenoid and granulomatous stomatitis, lichenoid lesion, Oral mucosa

## Abstract

**Background:**

Lichenoid and granulomatous stomatitis (LGS) is a rare condition, with clinicopathological features resembling lichenoid and granulomatous dermatitis. Despite increasing reports, its etiology remains unclear and detailed immunohistochemical (IHC) studies are lacking.

**Methods:**

We analyzed three new LGS cases, comparatively assessed with six cases each of oral lichen planus (OLP), oral lichenoid lesion (OLL), and oral leukoplakia (OL) showing lichenoid infiltrate. Histopathologic patterns were classified using the modified Magro & Crowson system, and IHC analysis included lymphocyte subsets, macrophages, and dendritic cells. Statistical comparisons were performed using Kruskal–Wallis with Dunn's post hoc test.

**Results:**

All LGS cases showed lymphohistiocytic infiltrates, microgranulomas, and perivascular/perineural lymphohistiocytic aggregates. IHC analysis revealed that LGS showed a significantly larger amount of macrophages (CD11c+/CD68+/CD163+) indicating M1/M2 equilibrium, CD3+ and CD8+ T‐cells, FOXP3+ regulatory T‐cells, and submucosal dendritic cells (FXIIIA+/CD209+) when compared to OLP/OLL/OL. Langerhans cells (CD1a+/CD207+) were similarly observed, whereas CD20+ B‐cells were scarce.

**Conclusions:**

This study provides the first comprehensive IHC analysis of LGS, highlighting distinctive macrophage, T‐cell, and dendritic cell profiles. These findings contribute to understanding LGS pathogenesis, support its diagnostic distinction, and suggest potential immunotherapeutic targets.

Lichenoid and granulomatous stomatitis (LGS) was firstly described by Robinson et al. in 2006 [1], which reported 6 cases, preferentially affecting the upper lip and adjacent gingiva of adult females, and whose clinicopathological features are similar with lichenoid and granulomatous dermatitis [2]. To date, about 65 cases of LGS have been reported [1, 3, 4, 5, 6, 7, 8]. Of them, 20 were males and 45 were females, with a mean age of 64 years (range, 30–88 years). The sites more affected were the upper labial mucosa and adjacent gingiva, followed by buccal mucosa, vestibule, and tongue. Clinically, LGS may mimic oral lichen planus (OLP), oral lichenoid lesion (OLL), oral leukoplakia (OL), squamous cell carcinoma, vesiculobullous lesions, trauma‐associated and allergy lesions [6]. Microscopy shows three distinct components, including lichenoid inflammation, granulomatous inflammation (epithelioid macrophages and/or giant cells) and lymphoid follicles/aggregates in perivascular and perineural distribution [6, 7]. Its etiology remains unknown, although a drug‐induced reaction was initially suggested [1]. Noteworthy, although some histiocytic and lymphoid markers assessing LGS were reported [1, 6], there is lack of a detailed immunohistochemical (IHC) study, which can aid in a better understanding of its pathogenesis with therapeutic and prognostic implications.

Here, we report a detailed clinicopathological and IHC analysis of 3 additional LGS cases, which, relevantly, were comparatively assessed with 6 cases each of OLP, OLL, and OL (microscopically exhibiting a lichenoid infiltrate). For LGS, histopathologic patterns followed the modified Magro & Crowson classification system [2, 6]. The criteria for IHC analysis of the lymphocyte populations, macrophages, and dendritic cells (DCs), were previously defined by us [9, 10]. This study was approved by the Ethics Committee in Human Research from FOAR/UNESP, Brazil (statement number: 64210122.7.0000.5416).

The Case 1 was a 50‐year‐old woman who presented with complaints of oral lesions and burning sensation several months ago. Her medical history revealed cervical carcinoma. Intraoral examination showed irregular and diffuse erythematous lesions in the upper labial mucosa and vestibular gingiva at level of teeth #13 to #24. The clinical differential diagnosis included OLP, OLL and cicatricial pemphigoid (Figure 1).

**FIGURE 1:**
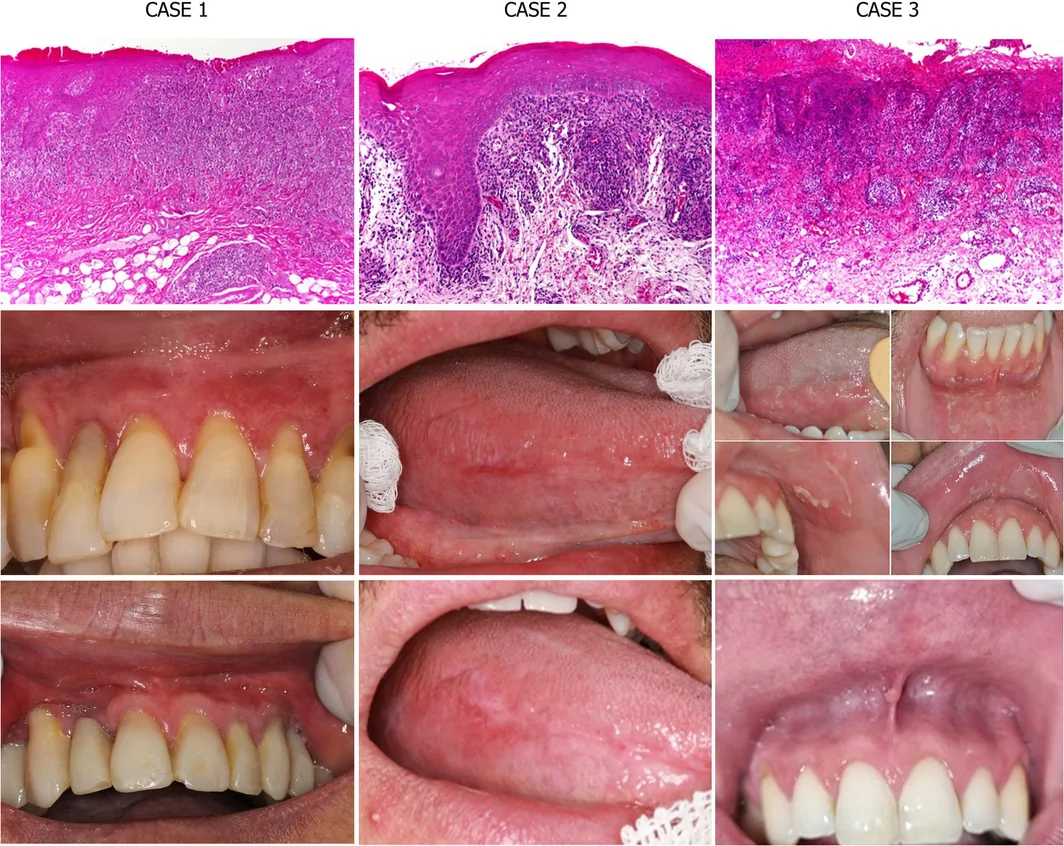
Representative clinical images and histopathological features. Top line showing slightly hyperplastic surface epithelium and lymphohistiocytic infiltrate with scarce microgranulomas in the lamina propria and submucosa. Notice the nodular growth pattern (H&E stain; × 100 [Case 1]; × 400 [cases 2 and 3]). Middle line showing the initial clinical features. Bottom line showing the clinical features after the treatment with topical corticosteroids.

The Case 2 was a 39‐year‐old man who was referred complaining of a symptomatic tongue in the last 3 months. Medical history and extraoral examination were unremarkable. Intraoral examination evidenced an irregular erythematous lesion with subtle whitish striations located on the right lateral border of the tongue. OLL and hairy leukoplakia were considered in the clinical differential diagnosis (Figure 1).

The Case 3 was a 36‐year‐old man referred presenting painful oral lesions several months ago. Medical history and extraoral examination were noncontributory. Intraoral examination revealed diffuse, irregular, yellowish‐white plaques and streaks, associated with ulcerations, involving the lips, buccal mucosa, tongue, and gingiva. The clinical differential diagnosis considered OLP, pemphigus vulgaris, and cicatricial pemphigoid (Figure 1).

In all these cases, an incisional biopsy was performed, and microscopy exhibited similar histopathological features, which showed the presence of slightly hyperplastic surface epithelium in close association with lymphohistiocytic infiltrate and scarce microgranulomas, supported by lamina propria containing dilated and congested small blood vessels. In the deep lamina propria and submucosa, lymphohistiocytic aggregates in perivascular and perineural growth pattern were visualized. Moreover, microulcerations alternating with areas of hyperkeratosis (Case 3) and overt basal cell degeneration admixed with apoptotic bodies (Cases 1 and 3) were also detected (Figure 1). Multinucleated giant cells and necrosis areas were not visualized. Histochemical analysis (Gram, periodic acid‐Schiff, Gomori methenamine silver, Warthin‐Starry, Fite, and Ziehl‐Neelsen stains) and anti‐ *Treponema pallidum* by IHC analysis, were all negative. Also, polarized light microscopy did not detect any foreign bodies. According to the modified Magro & Crowson classification system [6], all cases were Type 1.

In all three cases, treatment with topical corticosteroids was administered, showing improvement of the symptoms (all cases), as well as complete (Cases 1 and 3) and partial (Case 2) resolution of the lesions (Figure 1). No recurrences were detected after 3‐year of follow‐up.

Aiming at a comparative IHC analysis, 6 cases each of OLP (5 females, 1 male; mean age, 60 years), OLL (3 females, 3 males; mean age, 53 years), and OL (4 males, 2 females; mean age, 63 years), following strict diagnostic criteria [9, 10], were included (Figure 2). The results obtained were submitted to statistical analysis [9, 10].

**FIGURE 2:**
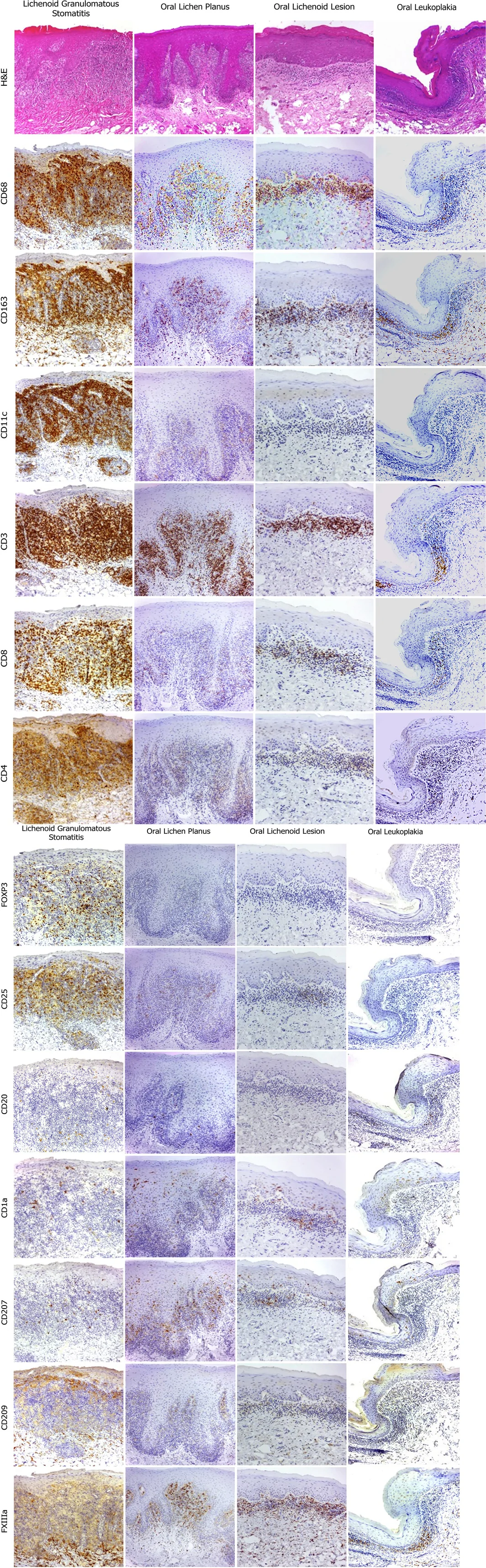
Histopathological and immunohistochemical findings. Through consecutive or serial histological sections, the microscopic (H&E stain) and immunohistochemical characteristics are shown in detail. Notice that CD11c, CD68, and CD163 markers highlighted an exuberant macrophage population, suggesting an equilibrium between M1 (CD11c+) and M2 (CD163+) macrophage populations. Similarly, a prominent CD3+ T‐cell population, permeated by numerous cytotoxic CD8+ T‐cells and CD4+ T‐cells, in LGS than OLP/OLL/OL were visualized (all figures, × 100). Immunohistochemical findings. Unlike from OLP/OLL/OL, notice that the uniform expression of FOXP3 and CD25 supports a distinctive regulatory T cell (Treg) population in LGS. CD20+ B‐cells were scarcely observed. Langerhans cells (CD1a+/CD207+) were slightly more frequent in OLP/OLL than LGS, followed by OL. Differently, FXIIIA+ and CD209+ cells were more evident in LGS when compared to OLP/OLL, and scarcely detected in OL (all figures, × 100).

Kruskal–Wallis with Dunn's post hoc test revealed significant differences when comparing the groups for all markers. In the current LGS cases, CD11c, CD68, and CD163, showed an exuberant macrophage population, when compared to OL (*p* = 0.0006) and OLP/OLL (*p* < 0.05), suggesting a M1 (CD11c+)/M2 (CD163+) macrophage equilibrium [11]. Similarly, a prominent CD3+ T‐cell population, permeated by numerous cytotoxic CD8+ T‐cells, when compared to OLP/OLL/OL (all, *p* = 0.0001) was detected. Also, CD4+ cells, probably representing Th cells and macrophages, were homogeneously distributed, being more numerous that OLP/OLL (*p* < 0.05) and OL (*p* = 0.0001) (Figure 2). Of note, the uniform expression of CD25 and FOXP3 supported a distinctive regulatory T cell (Treg) population; similarly, these cells were more frequently observed that OLP/OLL/OL (all, *p* = 0.0005). CD20+ B‐cells were scarcely observed. Thus, overall, the macrophage and lymphocytic populations above assessed were much more evident in LGS when compared to other groups. Considering DCs, Langerhans cells (CD1a+/CD207+) were slightly more frequent in OLP/OLL than LGS, followed by OL, with no statistical differences between them. Differently, submucosal DCs, including FXIIIA+ and CD209+ cells, were significantly more evident, with distinctive compartmentalization, in LGS when compared to OLP/OLL (*p* < 0.05) and OL (*p* = 0.0001) (Figure 2 continuation). The results of the statistical analysis are shown in Figure 3.

**FIGURE 3:**
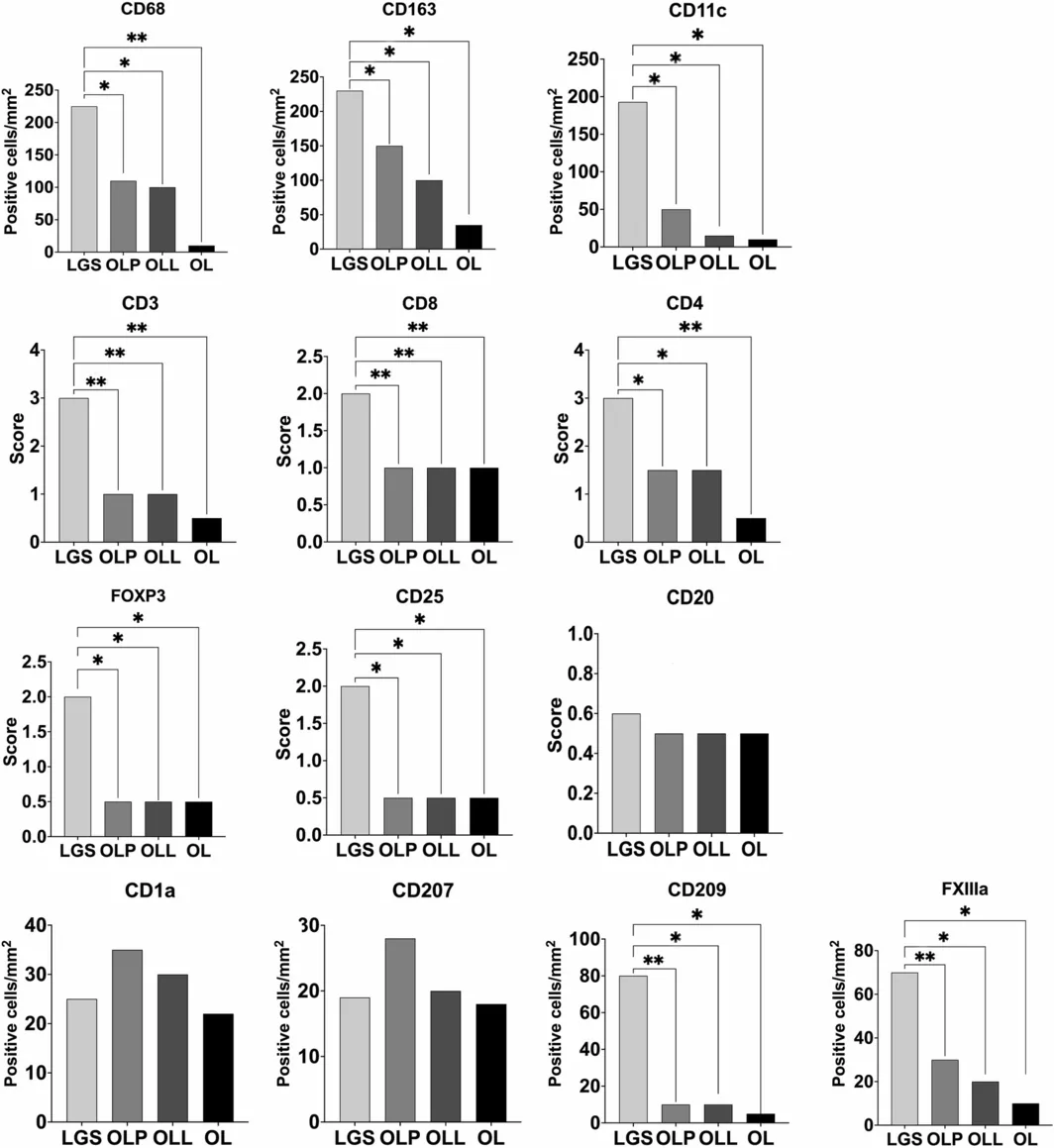
Quantitative analysis of the immune cell markers in LGS, OLP, OLL, and OL. Comparisons among groups were performed using the Kruskal–Wallis test followed by Dunn's multiple comparisons test. Significant differences are indicated by brackets and asterisks (*p < 0.05, **p ≤ 0.0006). The graphs were created using Prism 11 software.

Relevantly, only few studies have assessed LGS through CD3, CD4, CD8, CD20, and CD68 immunomarkers [1, 6]. Thus, to our knowledge, this is the first study showing a comprehensive IHC study in LGS. Our findings suggest that M1/M2 macrophage equilibrium, admixed with cytotoxic CD8+ T‐cells and Tregs, in close association with a distinctive compartmentalization of submucosal DCs, were notably more evident in LGS than OLP/OLL/OL, providing evidence that may aid in understanding their pathogenesis, with relevant diagnostic implications, as well as projecting potential immunotherapeutic targets.

## Funding

Andreia Bufalino (2022/14672‐0), Mariana Paravani Palaçon (2022/14734‐5), and Jorge Esquiche León (2022/12760‐9) received research grants from the São Paulo Research Foundation (FAPESP). Jorge Esquiche León received a research grant (304241/2021‐0) from the National Council for Scientific and Technological Development (CNPq).

## Ethics Statement

All procedures performed in studies involving human participants were in accordance with the ethical standards of the institutional and/or national research committee and with the 1964 Helsinki declaration and its later amendments or comparable ethical standards.

## Consent

Consent for publication was obtained for every individual person's data included in the study. This study was approved by the Ethics Committee in Human Research from UNESP (statement number: 64210122.7.0000.5416). Informed consent was obtained from all individual participants included in the study.

## Conflicts of Interest

The authors declare no conflicts of interest.

## Data Availability

Data sharing not applicable to this article as no datasets were generated or analysed during the current study.
